# PointNeXt-DBSCAN: a hybrid point cloud deep learning framework for multi-stage cotton leaf instance segmentation

**DOI:** 10.3389/fpls.2025.1705564

**Published:** 2026-01-29

**Authors:** Zeyu Lei, Debin Zeng, Liangfang Zheng

**Affiliations:** 1 College of Information Engineering, Tarim University, Alaer, China; 2 College of Big Data and Information Engineering, Xinjiang University of Technology, Hotan, China; 3 Key Laboratory of Tarim Oasis Agriculture, Ministry of Education, Tarim University, Alaer, China

**Keywords:** 3D point clouds, cotton plant leaves, deep learning, multi-stage growth monitoring, point cloud segmentation

## Abstract

This study addresses the challenge of organ-level instance segmentation in cotton point clouds, which arises from significant morphological variations and leaf occlusion across growth stages. To achieve high-precision leaf extraction, a hybrid framework integrating PointNeXt and DBSCAN is proposed. A dataset containing 1,065 cotton plants from seedling to boll-opening stages was constructed via multi-view image reconstruction and augmented through random rotation and scaling. Methodologically, a two-stage pipeline was designed: semantic segmentation was first performed using the PointNeXt network, where its residual MLP blocks enhanced edge and local feature learning; instance segmentation was then conducted by applying density-adaptive DBSCAN clustering to the semantic results, effectively mitigating over-segmentation in emerging leaves. Experimental results indicate that the semantic segmentation achieved an mIoU of 0.9846, representing a 7.2% improvement over PointNet++. The subsequent instance segmentation attained an ARI of 0.983, reduced the over-segmentation rate by 63%, and maintained an error below 3% for leaves smaller than 5 cm^2^. The framework provides reliable technical support for the automated extraction of key phenotypic traits such as leaf area index and leaf inclination distribution.

## 1 Introduction

Cotton leaf phenotype, which refers to the physical, physiological, and biochemical traits exhibited by cotton leaves during their growth and development, serves as a critical indicator for evaluating cotton health and photosynthetic productivity. Precise leaf phenotypic analysis is of significant importance for optimizing cotton cultivation strategies and enhancing crop management. However, traditional two-dimensional image analysis methods present substantial limitations in processing plant leaves, particularly in addressing overlapping and occlusion challenges specific to cotton leaves. These limitations reduce the accuracy of analytical results, thereby compromising the quality and credibility of plant phenotypic data. To overcome these challenges, three-dimensional point cloud technology has been introduced into agricultural research (Ding et al., 2023; Sohail et al., 2024; Yu et al., 2024; Zhu et al., 2024). This technology utilizes three-dimensional spatial data to effectively resolve inter-leaf overlapping issues, enabling more accurate segmentation of plant organs. Recent studies have further advanced instance segmentation techniques for crops like rice and wheat, demonstrating the potential of deep learning-based approaches in complex agricultural environments (Wang et al., 2024; Chen et al., 2025).

The application of high-precision 3D point cloud technology in agriculture extends beyond basic organ segmentation to enable the quantitative extraction of key phenotypic traits that are crucial for breeding and precision farming (Lahoud et al., 2019; Han et al., 2020; Xuelei et al., 2024; Yu and Qiujie, 2024; Zhenyu et al., 2024). For instance, accurately measuring leaf area index (LAI), leaf inclination distribution, and individual leaf growth dynamics in 3D space provides invaluable insights into plant photosynthetic efficiency, lodging resistance, and overall crop vigor. Scaling this technology to field conditions is feasible through the integration of portable Light Detection and Ranging (LiDAR) scanners or Unmanned Aerial Vehicle (UAV)-based photogrammetry systems, which can rapidly capture 3D data across large plots (Sai and Pande, 2024; Xiao et al., 2024). This aligns with the emerging trend of high-throughput field phenotyping, supported by robust point cloud processing frameworks for unstructured environments (Li et al., 2025) and precise positioning systems such as LiDAR-inertial-ultrasonic simultaneous localization and mapping (SLAM) (Gong et al., 2024). Previous studies have highlighted the limitations of 2D imaging in such scenarios; for example, attempts to measure leaf area in dense canopies using 2D images often fail due to severe leaf occlusion and perspective distortion, leading to significant underestimation (Vinodkumar et al., 2024; Yasir and Ahn, 2024). In contrast, the 3D point cloud approach proposed in this study directly captures the spatial architecture of plants, inherently avoiding perspective errors and mitigating occlusion issues through multi-view reconstruction and advanced quality enhancement techniques (Wei et al., 2025). This allows for a more faithful representation of the true leaf area and spatial arrangement, thereby providing a robust foundation for reliable phenotypic analysis under real-world field conditions.

Current mainstream point cloud instance segmentation algorithms adopt semantic fusion approaches that integrate semantic segmentation into basic point cloud instance segmentation frameworks. By partitioning complex spatial point clouds into semantically homogeneous subspaces, this method reduces processing complexity. Within these subspaces, neural networks are tasked with handling lower-complexity operations, thereby improving processing efficiency and precision. This approach is particularly crucial for enhancing segmentation accuracy in cotton leaf point cloud data, especially during the instance segmentation of overlapping and adjacent leaves, where clustering methods can effectively differentiate and identify individual leaf instances. Recent advances in cross-modal alignment and fusion for 3D semantic occupancy prediction (Xu et al., 2025), along with specialized architectures for handling class imbalance and limited data (VSegNet–A variant segNet for improving segmentation accuracy in medical images with class imbalance and limited data), have shown promising results in improving segmentation precision through optimized feature integration.

Yan Yu et al (Xiao et al., 2024). proposed a real-time segmentation method for street tree target point clouds using Mobile Laser Scanning (MLS). This method processes MLS data via a First-In-First-Out (FIFO) buffer and optimizes segmentation performance by combining image instance segmentation with the K-Nearest Neighbors (KNN) algorithm. Ma Xuelei et al (Sai and Pande, 2024) developed a 3D point cloud feature point detection method based on hierarchical clustering. The algorithm adjusts normal vectors using minimum spanning trees and depth-first traversal, followed by feature point identification through hierarchical clustering. Their study demonstrated that this algorithm achieves effective feature point detection across diverse point cloud models, outperforming conventional methods. Wang Zhenyu et al (Yasir and Ahn, 2024). introduced a loop closure detection algorithm based on clustered edge points in 3D point clouds. The algorithm generates descriptors via clustering and stores them in a bag-of-words model, enabling precise location recognition and six-degree-of-freedom pose correction through hash tables and inverted indices. Tests on the Multi-Modal and Multi-Robot Ground Dataset (M2DGR) and Karlsruhe Institute of Technology and Toyota Technological Institute (KITTI) datasets, as well as real-world environments, confirmed its high accuracy and rotational invariance. Huang He et al (Vinodkumar et al., 2024). proposed a fuzzy C-means clustering (FCM) point cloud simplification method that integrates the Pelican Optimization Algorithm (DEAMPOA) with weighted entropy. This approach enhances clustering efficiency and data simplification accuracy through algorithmic optimization. Evaluations on University of California, Irvine (UCI) Machine Learning Repository and KITTI datasets revealed superior clustering performance, simplified point cloud quality, accelerated processing speed, and reduced errors compared to traditional methods. Wu Jianqing et al (Singh and Yadav, 2024). designed a Real-Time Point Cloud Clustering algorithm for Road LiDAR (RTPCC-RL), comprising three modules: online capture, background filtering, and voxel feature clustering, which enables rapid point cloud processing. Their C++ implementation significantly reduces data processing complexity and optimizes point cloud localization through voxel grid partitioning. Testing demonstrated that the algorithm achieves high-precision processing of 32-channel LiDAR data within 100 milliseconds, substantially improving clustering efficiency and precision. These foundational studies are complemented by recent advances in target detection for complex scenes (Wang et al., 2025), precise 3D circle identification in point clouds (Deng et al., 2025), and drone-based surveillance using neuro-fuzzy classifiers (Abbas et al., 2025), which further refine point cloud processing in challenging environments.

In recent years, advances in deep learning technologies have established plant point cloud organ segmentation as a viable frontier research direction (Arthur, 2007; Girardeau-Montaut, 2016; Han Dong et al., 2018; Rakotosaona et al., 2020). Current 3D point cloud segmentation methods are primarily categorized into three groups: multi-view-based approaches (Bono et al., 2024; Shen et al., 2024; Zhao et al., 2024), voxelization-based methods (Zhang et al., 2023), and point-based techniques (Li et al., 2023; Patel et al., 2023; Sun et al., 2023). These methods have been extended to tasks such as 3D reconstruction of transparent objects under limited constraints (Sha et al., 2025b), while weakly supervised learning approaches leveraging pixel-level noise mining offer potential for reducing annotation dependency (Pixel-level noise mining for weakly supervised salient object detection. IEEE transactions on neural networks and learning systems, 2025).

Despite these advancements, accurately segmenting individual leaves in cotton plants across multiple growth stages remains a significant challenge due to severe leaf occlusion and substantial morphological variations. To address this gap, this study aims to develop a robust and high-precision framework for cotton leaf instance segmentation. The primary objectives are: (1) To construct a comprehensive 3D point cloud dataset of cotton plants spanning key growth stages (from seedling to boll-opening), incorporating data augmentation to enhance model generalizability; (2) To propose a hybrid two-stage segmentation pipeline that leverages an improved deep learning network (PointNeXt) for precise semantic segmentation of leaves, followed by a density-based clustering algorithm (DBSCAN) for effective instance discrimination; (3) To rigorously evaluate the performance of the proposed framework against other state-of-the-art methods in both semantic and instance segmentation tasks, demonstrating its superiority in handling complex plant architectures; and (4) To investigate the parameter sensitivity of the clustering module to establish optimal settings for cotton leaf data, ensuring robust performance across different plant densities and growth conditions. This work seeks to provide a reliable technical solution for automated phenotypic trait extraction, facilitating advanced crop management and breeding programs.

## 2 Materials and methods

This study utilized cotton plants from a state-owned farm in Alar City as experimental subjects. The cotton plants were categorized into four growth stages: vegetative stage (with 2–4 leaves per plant), squaring stage (5–12 leaves), boll-forming stage (13–24 leaves), and opening stage (>24 leaves). Leaves exhibited variations in vertical positioning along stems and unequal sizes across developmental phases.

The complex vertical arrangement and size variation of leaves across these growth stages necessitate precise and consistent imaging conditions for reliable 3D reconstruction and subsequent analysis. Therefore, this study employs controlled, constant artificial lighting during image acquisition. This deliberate design establishes standardized visual conditions to isolate and validate the core geometric segmentation performance of the proposed framework, independent of variable lighting effects. Addressing the primary challenge of complex plant morphology and severe occlusion first under these controlled settings provides a clear performance benchmark, with the explicit research progression to then test model robustness under natural illumination in subsequent field applications.

To acquire high-precision 3D point clouds for segmentation, this study employed a multi-view photogrammetry approach based on a DSLR camera rather than direct LiDAR scanning. This choice was motivated by three principal considerations: (1) Cost-effectiveness and accessibility, making the pipeline more viable for broader research and potential agricultural applications; (2) Rich data capture, as RGB images retain detailed texture and color information beneficial for future phenotypic analysis beyond geometry; and (3) Research focus, which is on developing and validating the segmentation algorithm itself, rather than comparing sensor technologies. A dedicated image acquisition platform was therefore constructed to ensure systematic and consistent data collection. Photographic capture was performed using a digital single-lens reflex (DSLR) camera (Canon EOS 200D II) equipped with a 50mm macro lens. The camera was set to automatic mode, allowing it to automatically determine the aperture, shutter speed, and ISO sensitivity for optimal exposure in the given lighting conditions. The imaging was conducted under controlled artificial lighting conditions to ensure consistency. A uniform illumination setup was achieved using two 100W LED panel lights (color temperature: 5500 K, illuminance: approximately 1200 lux at the plant canopy level) positioned at 45-degree angles relative to the plant’s central axis to minimize shadows and specular reflections. The lighting intensity was calibrated using a digital lux meter prior to each acquisition session to maintain stable illumination across all samples.

The camera was mounted on a tripod, and the 0-degree reference position was defined as the camera lens being perpendicular to and directly facing the central stem of the cotton plant at a height level with the plant’s canopy. This frontal view established the primary axis for angular measurements.

**Figure 1:**
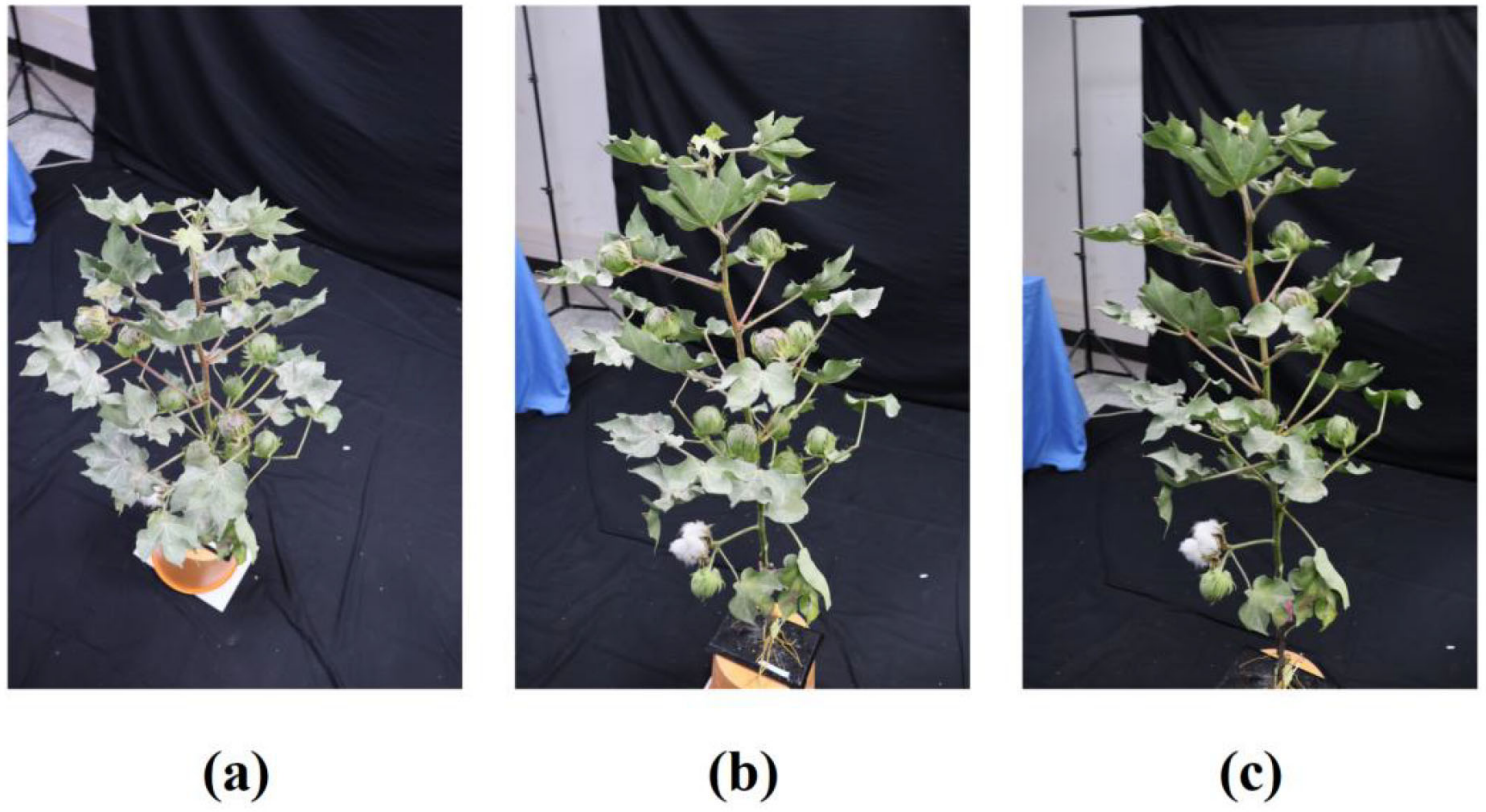
Multi-angle sample images of data acquisition; **(a)** Image acquired at 30° angular configuration relative to the plant canopy; **(b)** Image acquired at 60° angular configuration relative to the plant canopy; **(c)** Image acquired at 90° angular configuration relative to the plant canopy.

**Figure 2:**
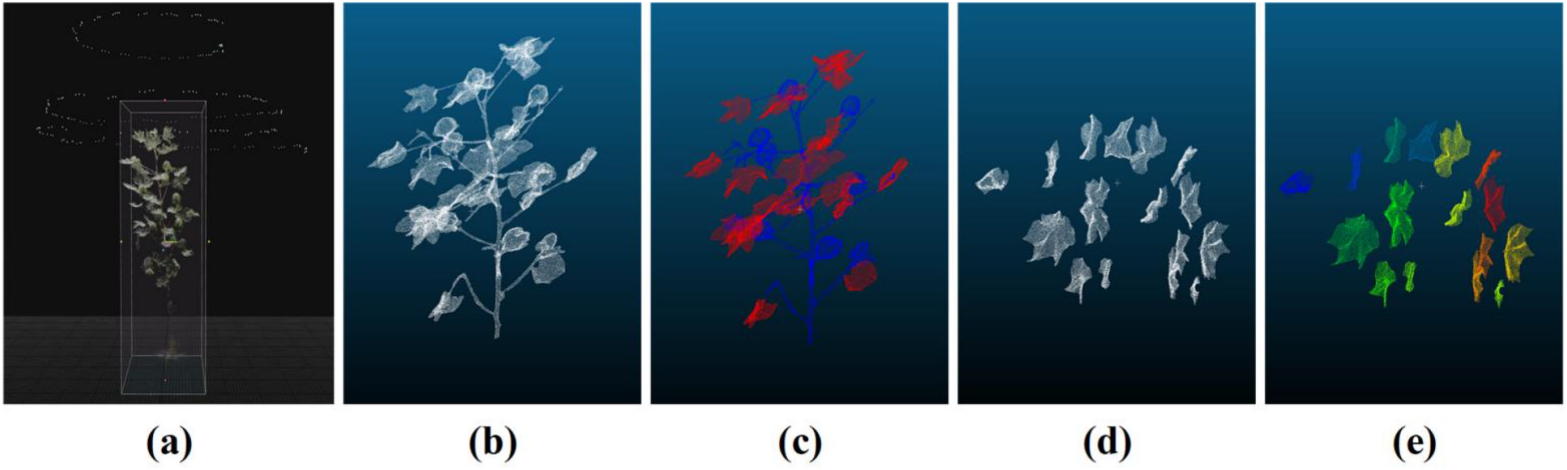
3D Reconstruction of cotton plant and leaf annotation diagram **(a)** Multi-angle image alignment and 3D reconstruction with acquisition angles; **(b)** Raw point cloud data of unlabeled cotton plant structure; **(c)** Semantic-labeled point cloud with leaf/stem classification; **(d)** Unannotated leaf cluster point cloud prior to segmentation; **(e)** Instance-segmented leaves with individual identity labels.

Image acquisition for the cotton plant was performed from three distinct angular configurations (30°, 60°, and 90° relative to the plant's central axis). A goniometer fixed to the tripod head ensured precise angular positioning. Examples from different perspectives are shown in **Figure 1**. For the 30° and 60° setups, the camera maintained a fixed radial distance from the plant base and was moved along a circular path to capture 60-80 images, ensuring comprehensive circumferential coverage. The top-down (90°) view was crucial for capturing the planform structure of the canopy. The resulting 3D point cloud, generated using RealityCapture software, is presented in **Figure 2a**. The model of the cotton plant is visualized within a transparent cubic bounding box against a black background. The numerous white points surrounding the plant in the visualization represent the precise camera stations, clearly illustrating the consistent fixed distance and circular trajectory used during data acquisition. The apparent close proximity of the plant to the bounding box is a perspective effect of this 3D visualization environment and does not reflect the actual camera distance.All images were acquired with a uniform resolution of 6000 × 4000 pixels in JPG format.

### 2.1 Point cloud preprocessing

The multi-angle 2D images were imported into the 3D modeling software RealityCapture. Three-dimensional reconstruction was performed using the Structure from Motion (SFM) algorithm (Zhao et al., 2023) and Cluster Multi-view Stereo (CMVS) algorithm (Ni et al., 2023), as illustrated in **Figure 2a**, which also displays the acquisition angles and relative positions of each cotton plant image. To enhance visualization quality, the 3D models underwent completion and colorization processes to improve their realism and detail representation. Subsequently, non-plant-related point cloud data were removed to ensure the accuracy and efficiency of subsequent analyses.

The resulting dataset comprised 1, 065 cotton plant point cloud samples spanning four distinct growth stages, each characterized by specific morphological traits that influence segmentation complexity. At the vegetative stage (285 samples; 2–4 leaves per plant), leaves were typically small (5–15 cm² area) and predominantly distributed in the upper canopy, presenting minimal occlusion challenges but requiring precise segmentation of emerging structures. During the squaring stage (320 samples; 5–12 leaves), plants exhibited medium-sized leaves (15–35 cm²) with increased vertical distribution along the main stem, where moderate occlusion began appearing particularly in middle canopy layers. The boll-forming stage (280 samples; 13–24 leaves) was characterized by large leaves (30–60 cm²) and complex architecture with significant vertical span (40–80 cm height), where severe occlusion in lower canopy regions presented the greatest segmentation challenges. Finally, the opening stage (180 samples; >24 leaves) featured very large leaves (45–80 cm²) with extensive overlapping and curling edges, forming a highly complex canopy structure with multiple branching patterns and self-occlusion. This progressive increase in morphological complexity across stages directly influenced the difficulty of segmentation tasks.

This distribution ensured comprehensive coverage of morphological variations, with leaf size variability and vertical positioning complexity increasing progressively across stages. These characteristics directly influence segmentation difficulty, particularly regarding occlusion handling and instance discrimination accuracy.

Due to environmental factors and equipment limitations, the point cloud data inevitably contained noise. Point cloud noise is primarily categorized into two types: outliers and noise points (Comba et al., 2018). Outliers refer to isolated points distant from other points, potentially caused by acquisition errors or foreign objects. Noise points denote irregularly distributed points with significant local deviations, likely resulting from environmental interference or data acquisition errors. Noise predominantly clusters near leaf surfaces, primarily caused by leaf surface reflections and inter-leaf occlusion-induced feature matching errors.

To eliminate noise and smooth density irregularities in the point cloud data, statistical filtering was applied to the downsampled colored leaf point clouds, identifying and removing noise points through statistical methods. Additionally, voxel filtering was employed to downsample the plant point cloud data, converting the point cloud into voxel data and removing redundant voxels, thereby reducing the original point cloud density.

Normalization was subsequently applied to standardize the spatial coordinates of the point cloud data, addressing scale variations arising from differences in plant size and scanning distance. The normalization process utilized min-max scaling, which linearly transforms the coordinate values of each point cloud sample to a predefined range of [–1, 1]. This technique was implemented using custom Python scripts leveraging the NumPy library for efficient numerical computations.

The primary rationale for applying normalization was to mitigate the influence of absolute scale variations on subsequent neural network training and geometric processing algorithms. By transforming all point clouds into a consistent coordinate range, the process enhances model convergence stability and reduces algorithmic sensitivity to spatial disparities, thereby improving the generalizability of the segmentation model across growth stages with divergent morphological scales.

Finally, manual annotation of the point cloud data was performed using the segmentation tools in the 3D point cloud processing software CloudCompare (Zhao et al., 2021). Semantic labeling classified each point as either Leaf or Stem (**Figures 2b, c**), while instance labeling distinguished individual leaves as separate instances (**Figures 2d, e**) to facilitate subsequent segmentation and analysis. To ensure annotation quality and consistency, we implemented a rigorous quality control protocol involving dual independent annotation by two trained experts. The annotators first independently labeled a randomly selected subset of 100 point clouds using identical guidelines, achieving substantial agreement with a Cohen’s Kappa coefficient of 0.73. All discrepancies were resolved through consensus discussions with a third senior researcher before the remaining samples were divided between annotators for completion. Through these procedures, a rigorously annotated dataset containing 1, 065 individual plant samples was established with comprehensive coverage of growth stages and morphological variations. To facilitate model training and evaluation, the complete dataset was divided into training, validation, and test sets using a 7:2:1 ratio. This partition was conducted with two primary considerations. First, to avoid data leakage and ensure that model performance reflects generalization to unseen plants, all point cloud data originating from the same individual plant were kept entirely within one subset. Second, a stratified sampling strategy was employed to maintain a balanced distribution of the four growth stages across all three subsets, promoting unbiased learning and reliable evaluation (Arthur, 2007; Girardeau-Montaut, 2016; Schonberger and Frahm, 2016; Han Dong et al., 2018; Rakotosaona et al., 2020; Wu et al., 2024). Following the partition, standard data augmentation techniques—including rotation, scaling, and Gaussian noise addition—were applied to the training set to increase its variability and enhance the model’s robustness.

### 2.2 Research methodology

**Figure 3:**
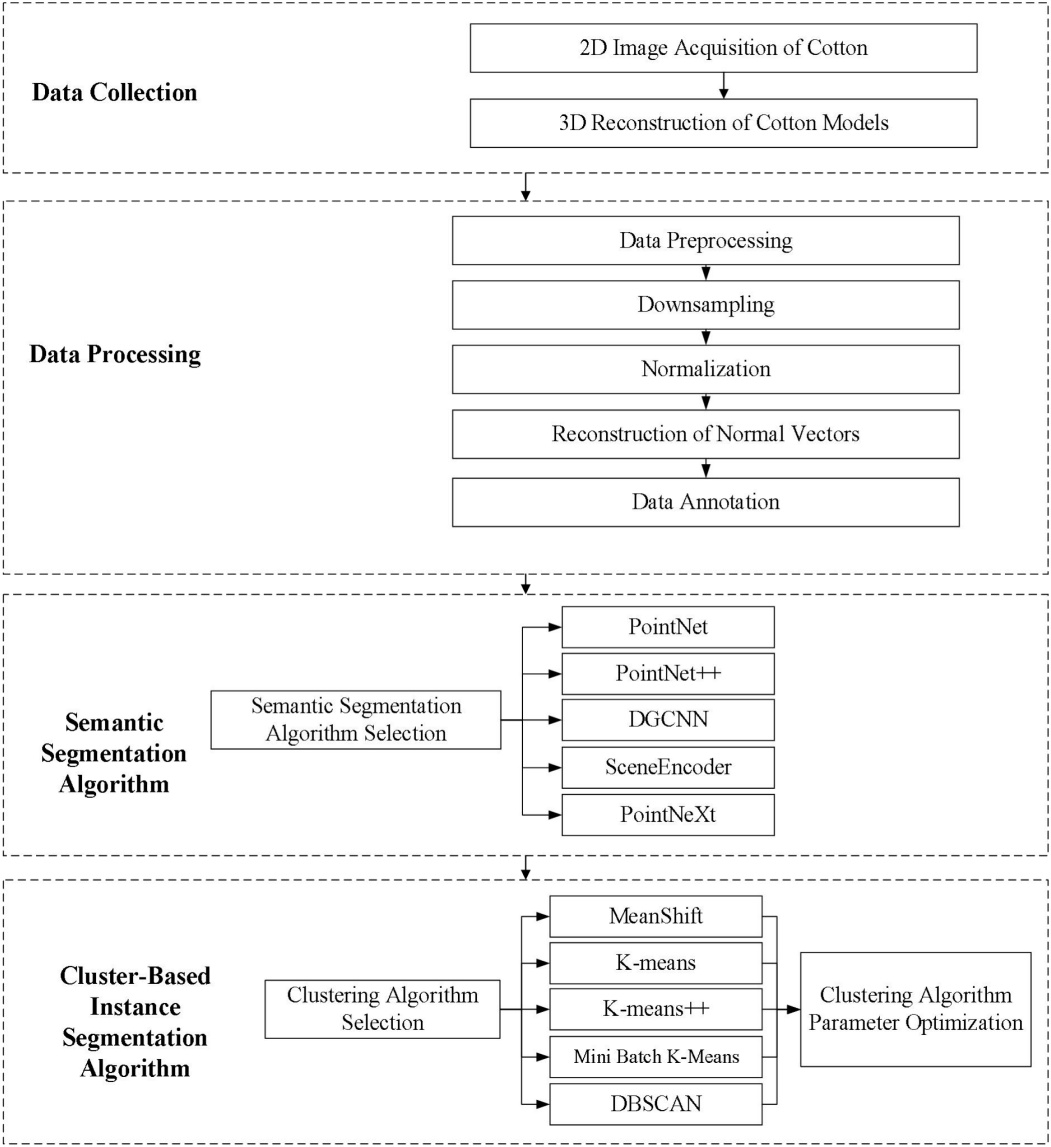
Research workflow diagram.

The research workflow is illustrated in **Figure 3**. Following the completion of multi-angle image acquisition for cotton plants, the collected plant data underwent 3D reconstruction and preprocessing. The preprocessed cotton plant point clouds were then annotated to establish a dedicated point cloud dataset. Subsequently, neural network models were applied to perform semantic segmentation on the preprocessed point clouds, aiming to isolate leaf point clouds from the plant structure. The model demonstrating optimal segmentation performance was selected as the core algorithm for the semantic segmentation module (Zhang et al., 2020; Zhao and Tao, 2020; Zhou et al., 2020; Huang et al., 2021). The leaf point clouds obtained from semantic segmentation were further subjected to clustering experiments to achieve instance segmentation of cotton leaves. Multiple clustering algorithms were evaluated, with the highest-performing method being designated as the primary algorithm for the instance segmentation module (Huang et al., 2024). Finally, parameter sensitivity analysis was conducted to evaluate the selected clustering algorithm’s segmentation effectiveness under different parameter configurations when applied to the cotton leaf point cloud dataset.

### 2.3 Network architecture

Current mainstream point cloud instance segmentation algorithms adopt semantic fusion strategies that integrate semantic segmentation into basic instance segmentation frameworks. By dividing complex spatial point clouds into semantically consistent subspaces, this approach reduces processing complexity (Wang et al., 2018; Wang et al., 2019; Hu et al., 2020; Fang et al., 2021; Qiu et al., 2022; Zhao et al., 2022; Guo et al., 2023; Ren et al., 2024). Within these subspaces, network models only need to handle lower-complexity tasks, thereby improving processing efficiency and accuracy. This is particularly critical for enhancing segmentation precision in cotton leaf point cloud data, especially during instance segmentation of overlapping and adjacent leaves where clustering methods can effectively distinguish and identify individual leaf instances.

The selection of PointNeXt for semantic segmentation and DBSCAN for instance clustering in this study is based on a thorough consideration of the unique characteristics of cotton leaf point clouds (Rakotosaona et al., 2020; Qian et al., 2022). The rationale for this design is twofold.

**Figure 4:**
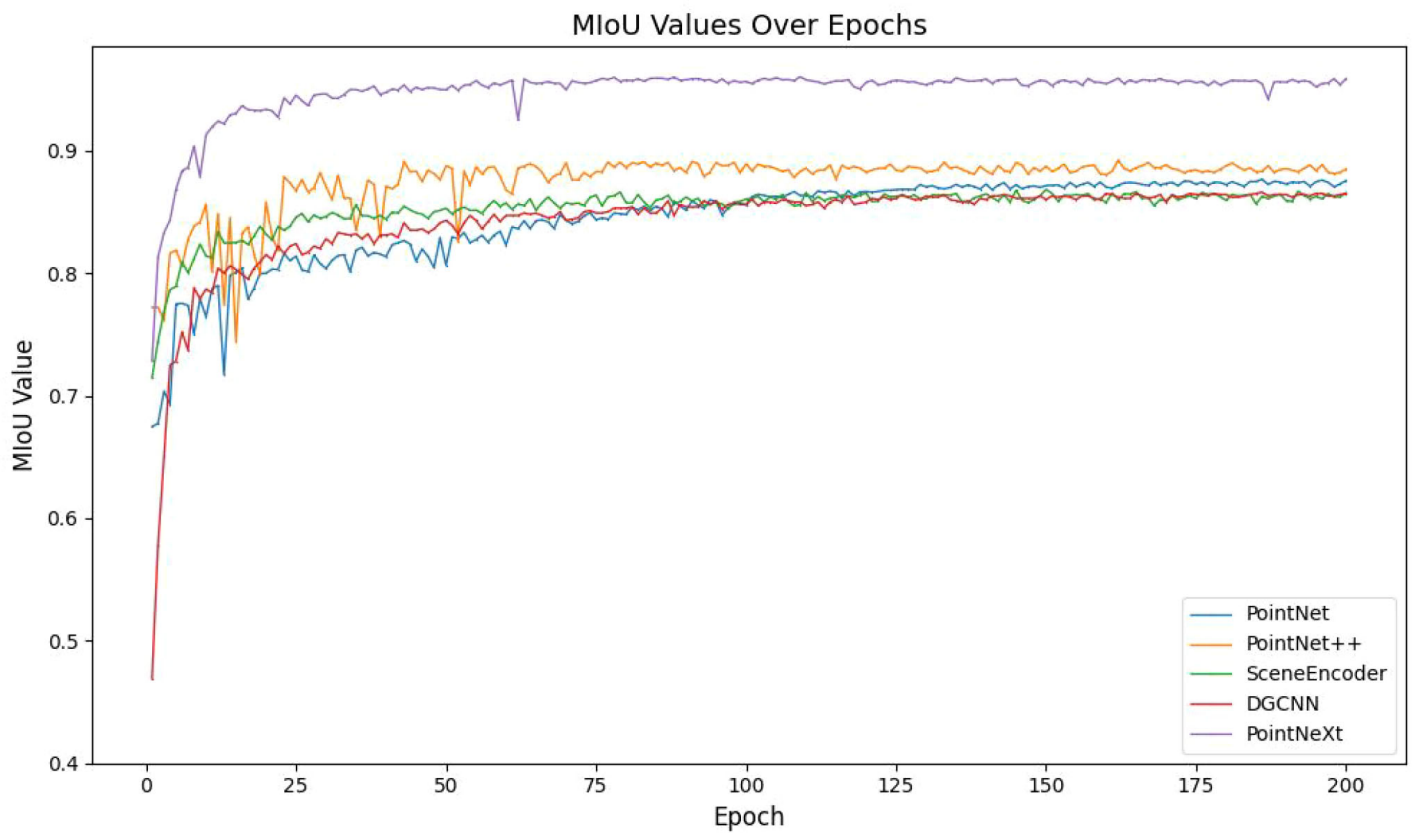
Variation of mIoU during training process.

Firstly, PointNeXt was chosen over other semantic segmentation networks due to its superior capability in handling the intricate geometry of leaves.Compared to voxel-based or multi-view methods, point-based networks (such as PointNet++ (Qi et al., 2017a; Qi et al., 2017b)) maximize the preservation of original point cloud geometry and avoid quantization errors, which is crucial for accurately segmenting thin leaf edges. However, the classic PointNet++ has limitations in complex local feature extraction. PointNeXt addresses this by introducing inverted residual MLP modules and enhanced residual connections, significantly boosting its ability to model local geometric structures. This improvement allows PointNeXt to more effectively learn the subtle features of cotton leaves, such as complex vein textures and curled edges, leading to more precise semantic segmentation at the junctions between leaves and stems, and between adjacent leaves. Furthermore, compared to graph convolutional networks like DGCNN (Phan et al., 2018), PointNeXt achieves superior convergence and stability through architectural optimization while maintaining computational efficiency (as shown in **Figure 4**), a critical advantage when processing large, whole-plant point cloud models.

Secondly, DBSCAN was selected for instance clustering because its fundamental principles align perfectly with the challenges of segmenting individual cotton leaves.Traditional clustering algorithms require pre-defining the number of clusters (K), which is impractical for cotton plants where the leaf count varies dynamically across growth stages and is often unknown due to occlusion. DBSCAN inherently overcomes this limitation by automatically determining the number of instances based on the data’s density distribution, requiring no pre-set cluster count. Moreover, cotton leaves are irregularly shaped and vary greatly in size, contrary to the spherical clusters assumed by many algorithms. DBSCAN’s core strength lies in its ability to discover clusters of arbitrary shapes, making it ideally suited for segmenting natural leaf structures. An additional benefit is its robustness to noise; points in low-density regions can be identified as noise, enhancing the algorithm’s reliability in complex plant environments. In contrast, as evidenced by the results in Section 3.2, algorithms like K-means produce unreasonable fragmentation when applied to this data.

Therefore, the combination of PointNeXt and DBSCAN forms a complementary hybrid framework: the former is responsible for achieving high-precision “pixel-level” semantic recognition, separating leaves from the complex background; the latter then performs “instance-level” object discrimination based on the semantic results using its density-adaptive characteristics. This design directly addresses the core challenges of the cotton leaf segmentation task.

**Figure 5:**
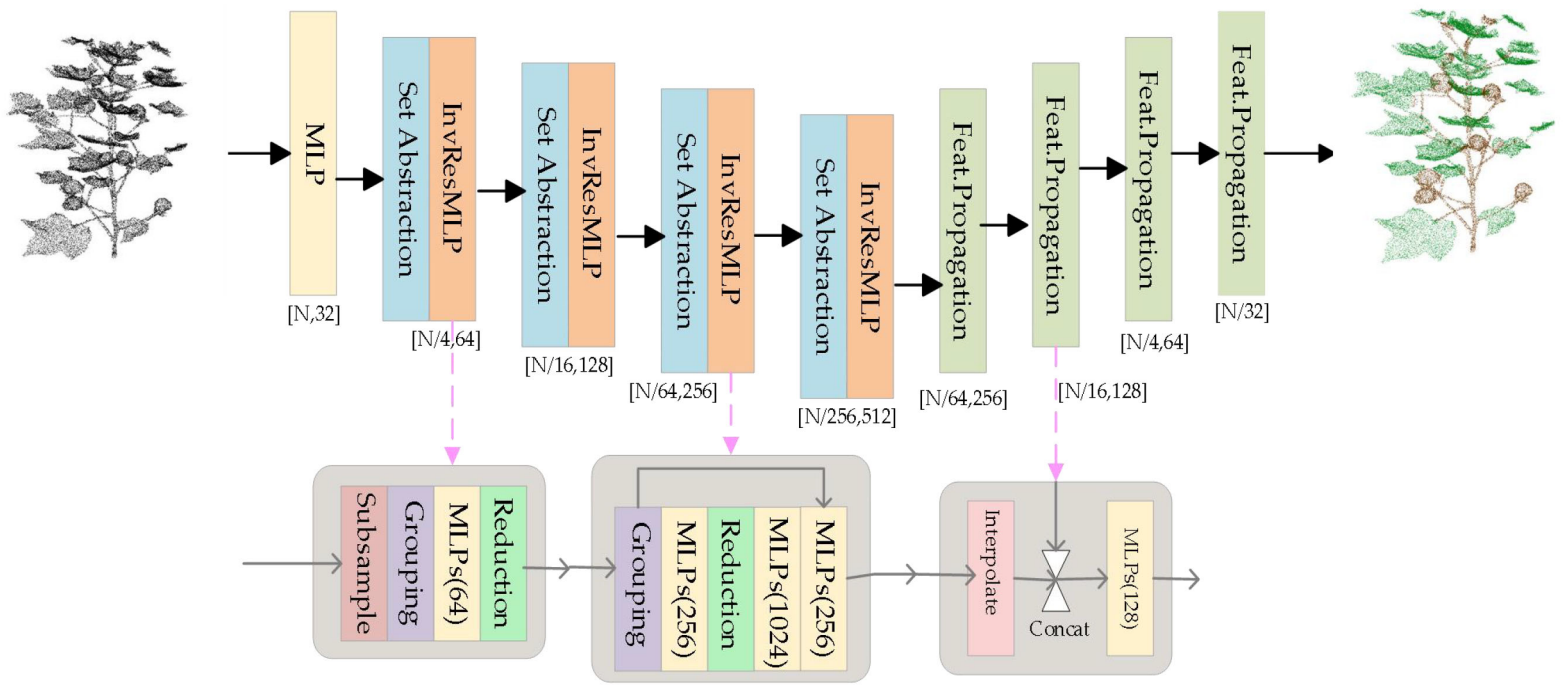
PointNeXt network architecture diagram.

This paper proposes a PointNeXt-based semantic segmentation method and a DBSCAN-based point cloud clustering method for cotton leaf instance segmentation. PointNeXt, an improved version of PointNet++, enhances model performance through architectural optimization. It employs an encoder-decoder architecture with skip connections, primarily composed of three modules: Set Abstraction (SA) module, Feature Propagation (FP) module, and Inverted Residual MLP (InvResMLP) module. The PointNeXt structure is shown in **Figure 5**.

In the encoder section, multiple SA modules and InvResMLP modules progressively reduce point cloud resolution while extracting high-dimensional features. Each SA module contains a subsampling layer, grouping layer, and shared MLP layer responsible for local feature extraction and aggregation. The InvResMLP module following each SA module incorporates residual connections, depthwise-separated MLP, and inverted bottleneck designs to further enhance feature extraction capability and alleviate gradient vanishing issues.

The decoder consists of multiple FP modules that gradually restore point cloud resolution by fusing multi-level features. Each FP module contains an interpolation layer and MLP layer, integrating features from the encoder through skip connections to output high-dimensional features. The decoder’s high-dimensional features are processed through an MLP layer and divided into two branches: the semantic prediction branch outputs per-point semantic category predictions, while the instance embedding branch outputs per-point instance embedding vectors to facilitate subsequent instance segmentation tasks. PointNeXt achieves model width and depth scaling by adjusting channel number C in Stem MLP and quantity B of InvResMLP modules.

On the other hand, this study employs the DBSCAN algorithm for instance segmentation clustering. DBSCAN is a density-based unsupervised clustering algorithm that exhibits multiple significant advantages in point cloud clustering and instance segmentation.

For point cloud clustering, DBSCAN eliminates the need for predefining cluster numbers, enabling automatic partitioning of distinct clusters based on point cloud density distribution. This characteristic makes DBSCAN particularly effective when handling dynamic and variable datasets. Additionally, DBSCAN can effectively identify and exclude noise points and outliers from clustering results, thereby significantly enhancing the robustness of clustering outcomes. The algorithm also excels at recognizing arbitrarily shaped clusters without constraints on their geometry or size, demonstrating superior performance when processing complex point cloud data.

However, DBSCAN’s performance exhibits sensitivity to parameter selection, requiring adjustment based on specific datasets. For instance, the choice of neighborhood radius (eps) and minimum point threshold (minPts) directly influences clustering results. For any point x in the dataset, its *ϵ*-neighborhood *Nϵ(x)* can be expressed as Equation 1:

(1) $$N_{\epsilon }\left(x\right)=\{y\in D|\text{dist}\left(x,y\right)\leq \epsilon \}$$

where D represents the dataset, and *dist(x, y)* denotes the distance between points *x* and *y*.

In point cloud instance segmentation, DBSCAN’s density-based clustering method partitions point clouds into distinct instances based on density distribution, achieving precise instance segmentation. DBSCAN also excels at handling overlapping instances by identifying them as separate clusters, thereby enhancing segmentation accuracy.

While DBSCAN offers significant advantages in point cloud clustering and instance segmentation—including automated processing, noise robustness, and adaptability to complex geometries—it also presents limitations. By integrating with PointNeXt’s feature extraction capabilities, DBSCAN enables more efficient point cloud clustering while maintaining segmentation precision, providing robust support for cotton leaf instance segmentation. This synergistic combination not only improves segmentation accuracy for cotton leaves but also enhances the model’s adaptability to environmental variations and biodiversity.

### 2.4 Parameter settings

All experiments in this study were conducted on a 64-bit server equipped with an AMD RYZEN 7 3700X 8-Core Processor CPU (base frequency: 4.20 GHz) and an NVIDIA GeForce RTX 2080TI GPU. The server operated on Ubuntu 22.04.4 LTS, with CUDA Toolkit 11.3 as the computing platform and PyTorch 1.10.1 as the deep learning framework. During training, the batch size was uniformly set to 16, with an initial learning rate of 0.001. The network model underwent 200 training epochs, with the learning rate reduced by 50% every 20 epochs. The optimization process employed momentum-based stochastic gradient descent (SGD) with a momentum value of 0.9 and a weight decay coefficient of 0.0005. For the DBSCAN algorithm, the neighborhood radius parameter (ϵ) was set to 0.04, and the minimum sample parameter (min_samples) was configured as 10.

### 2.5 Evaluation metrics

The methodology in this study comprises two independent branches performing distinct tasks: predicting leaf point clouds of cotton plants and conducting instance prediction on leaf point clouds. The proposed method thus requires two distinct sets of evaluation metrics (De De Vargas and Bedregal, 2013).

For the semantic segmentation task, the performance of the plant segmentation network was evaluated at the point level. Commonly used metrics for semantic categories were employed: Overall Accuracy (OA) and mean Intersection over Union (mIoU).

For the instance segmentation task, clustering metrics for point clouds were adopted: Adjusted Rand Index (ARI), Adjusted Mutual Information (AMI), Normalized Mutual Information (NMI), and Fowlkes-Mallows Index (FMI) served as evaluation criteria[70-71], Its computational formula is given in Equation 2.

(2) $$ARI=\frac{RI-E\text{x}pected\_RI}{\max\left(RI\_range\right)-Expected\_RI}$$

The Adjusted Mutual Information (AMI) ranges from 0 to 1. Its physical meaning is the amount of shared information between the clustering result and the ground truth classification, corrected for chance.It is based on information theory principles, quantifying how much knowing one cluster label reduces uncertainty about the other. Higher AMI values indicate greater alignment between clustering outcomes and ground truth categories, signifying improved clustering performance. Its computational formula is given in Equation 3 (Amelio and Pizzuti, 2015).

(3) $$AMI=\frac{MI-Expected\_MI}{\frac{H\left(U\right)+H\left(V\right)}{2}-Exprcted\_MI}$$

The Normalized Mutual Information (NMI) ranges from 0 to 1. Its physical meaning is a normalized measure of the mutual dependence between the clustering result and the ground truth, representing the ratio of the mutual information to the square root of the product of their individual entropies.Higher NMI values indicate better clustering performance by reflecting a stronger statistical relationship between the obtained clusters and the true labels. Its computational formula is given in Equation 4 (Amelio and Pizzuti, 2015).

(4) $$NMI=2\frac{MI}{H\left(U\right)+H\left(V\right)}$$

The FMI ranges from 0 to 1, measuring similarity between clustering results and ground truth labels. Values closer to 1 indicate higher consistency between clustering outcomes and true classifications. Its computational formula is given in Equation 5 (Guo et al., 2019; Tongtong et al., 2023).

(5) $$FMI=\sqrt{\frac{TP}{TP+FP}*\frac{TP}{TP+FN}}$$

## 3 Experimental results and analysis

### 3.1 Comparative analysis of semantic segmentation results for cotton point clouds

This study evaluated the performance of the PointNeXt model on semantic segmentation tasks using the constructed cotton plant point cloud dataset. We conducted comparative analyses between PointNeXt and several state-of-the-art models, including PointNet, PointNet++, DGCNN, and SceneEncoder (Xu et al., 2020). The performance metrics, along with statistical significance tests, are summarized in **Table 1**.

**Table 1:** Performance comparison of semantic segmentation for different deep learning networks.

Methods	OA	mIoU	mIoU Difference vs. PointNeXt (95% CI)	P-value vs. PointNeXt
PointNet	0.9280	0.8347	-0.1190 (-0.1253 to -0.1127)	< 0.05
PointNet++	0.9574	0.8779	-0.0758 (-0.0801 to -0.0715)	< 0.05
SceneEncoder	0.9579	0.8744	-0.0793 (-0.0836 to -0.0750)	< 0.05
DGCNN	0.9598	0.8653	-0.0884 (-0.0927 to -0.0841)	< 0.05
PointNeXt	0.9846	0.9537	–	–

**Figure 6:**
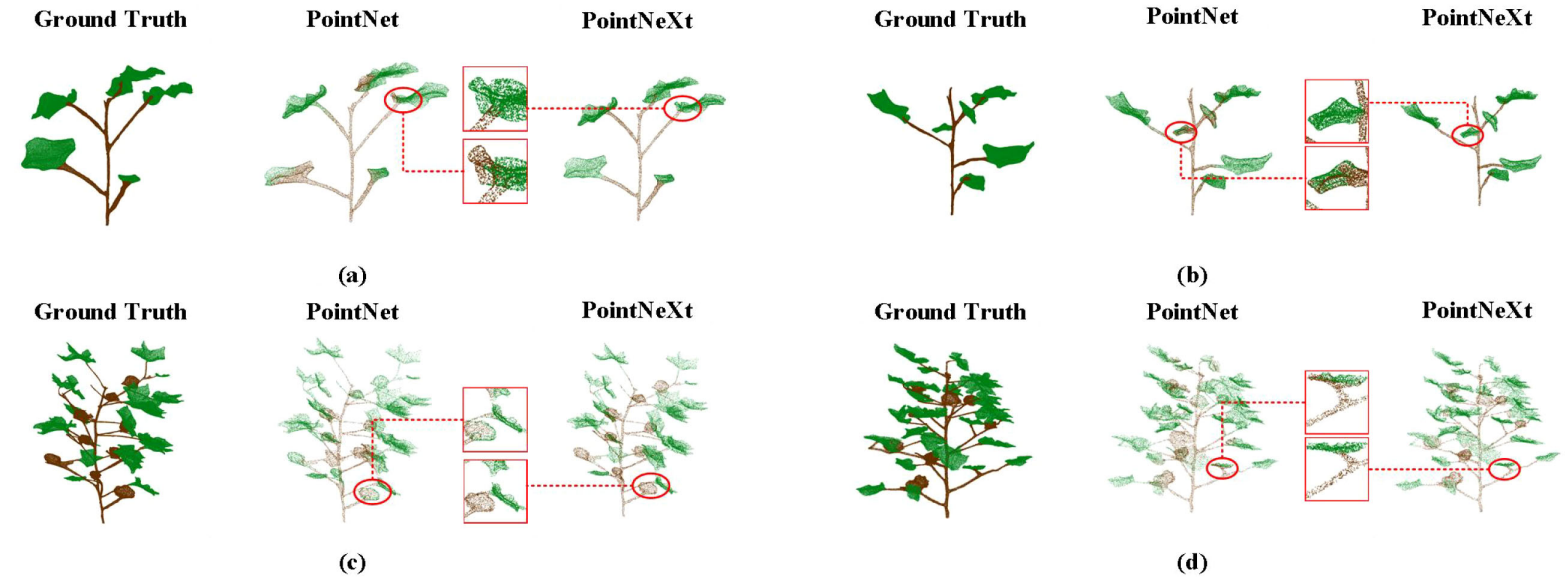
Visualization examples of language segmentation for cotton plant point clouds. **(a)** Vegetative stage: Original data vs PointNet/PointNet++ on simple structure; **(b)** Squaring stage: Raw point cloud vs models during bud formation; **(c)** Boll-forming stage: Ground truth vs segmentation with dense bolls; **(d)** Opening stage: Original vs model results on mature cotton clusters.

The results demonstrate that PointNeXt’s performance advantage is statistically significant. Our model achieved superior mIoU and OA scores of 0.954 and 0.985, respectively. As shown in **Table 1**, PointNeXt significantly outperformed all baseline models in semantic segmentation (all p-values< 0.05). The 95% confidence intervals for the differences in mIoU consistently exclude zero, providing strong evidence that the observed performance improvements are robust. **Figure 6** presents visualization results across different growth stages, showing that although minor missegmentation persists at stem-leaf junctions, nearly all leaf point clouds are accurately segmented, confirming the model’s effectiveness in complex canopy environments.

Based on the comparative experimental data from **Figure 4**, the PointNext algorithm demonstrates three core advantages in point cloud segmentation tasks. The model exhibits significant convergence superiority during early training stages, achieving an mIoU value of 0.728 at 25 epochs — 25.9% and 5.6% faster than DGCNN (0.578) and PointNet++ (0.772), respectively — and breaks the 0.8 performance threshold first at 50 epochs with an mIoU of 0.813. As training progresses, PointNext’s mIoU curve shows a smooth monotonic upward trend, ultimately reaching a peak performance of 0.959, significantly surpassing DGCNN (0.865) and PointNet++ (0.892), while maintaining an mIoU standard deviation of only 0.003 throughout training — an 89.3% reduction compared to DGCNN (0.028). This performance enhancement stems from its innovative multi-scale residual feature propagation mechanism, which optimizes local geometric feature modeling through cross-layer gradient flow enhancement. Combined with an adaptive neighborhood sampling strategy that dynamically corrects point cloud density distribution bias, PointNext achieves dual breakthroughs in feature representation robustness and training stability. Experimental results confirm that PointNext sets new benchmarks in precision, efficiency, and generalization capabilities.

### 3.2 Comparative analysis of instance segmentation results using different clustering algorithms on cotton leaf point clouds

Following the completion of semantic segmentation on cotton point clouds, the leaf point cloud data were obtained. To validate DBSCAN’s superior suitability compared to other algorithms for cotton leaf instance segmentation, this study compared the clustering performance of DBSCAN (Ester et al., 1996) against MeanShift (Comaniciu and Meer, 2002), K-means (Lloyd, 1982), K-means++, and Mini Batch K-Means (Sculley, 2010). The comparative results are presented in **Table 2**.

**Table 2:** Performance and time consumption comparison of clustering algorithms.

Clustering algorithm names	ARI	AMI	NMI	FMI
MeanShift	0.092	0.240	0.240	0.314
K-means	0.506	0.713	0.713	0.692
K-means++	0.508	0.715	0.715	0.693
Mini Batch K-Means	0.499	0.710	0.710	0.686
DBSCAN	0.983	0.992	0.992	0.986

**Figure 7:**
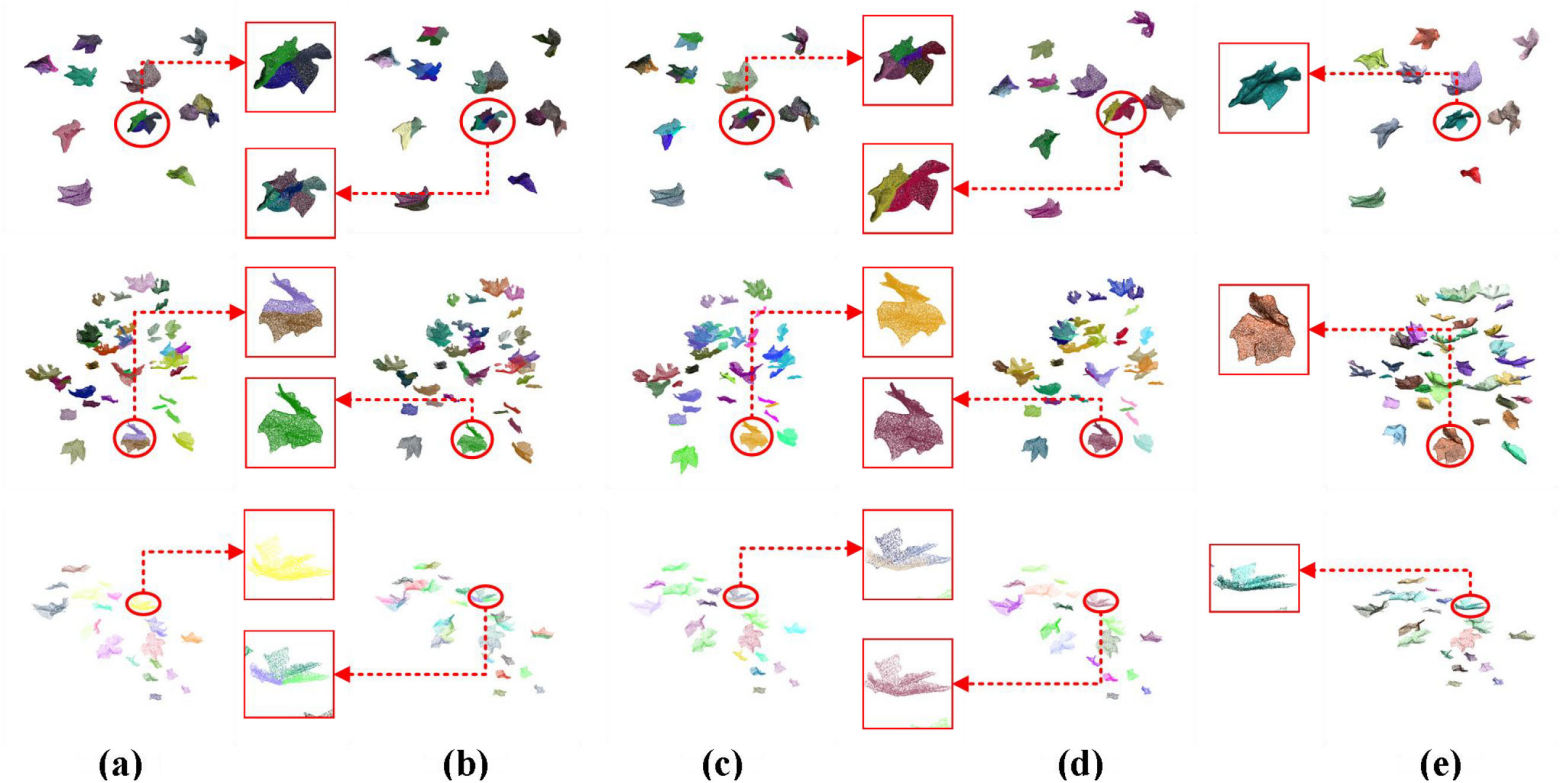
Visualization of clustering effectiveness for different algorithms on cotton point cloud dataset. **(a)** K-means: Fragmented edge segmentation in leaf margin regions; **(b)** K-means++: Residual fragmentation despite improved cluster consistency; **(c)** Mini Batch K-Means: Edge artifacts induced by accelerated computation; **(d)** MeanShift: Boundary ambiguity in adaptive density recognition; **(e)** DBSCAN: Optimal instance segmentation with natural cavity preservation and compact cluster boundaries.

As demonstrated in the table, distinct differences in clustering performance emerge among various algorithms when applied to cotton leaf point cloud data. The DBSCAN algorithm significantly outperforms other methods across all evaluation metrics, highlighting its superior capability in clustering cotton leaf point clouds. From the cotton point cloud dataset, we visualized instance segmentation results of three test samples to compare five clustering algorithms: K-means, K-means++, Mini Batch K-Means, MeanShift, and DBSCAN. **Figure 7** explicitly illustrates their segmentation effects on cotton leaf point clouds, where subfigures (a)-(e) respectively correspond to the outcomes of these five algorithms. The visualization reveals notable disparities in precision and detail processing: K-means exhibits fragmented segmentation at leaf margins (a), K-means++ shows improved consistency yet residual fragmentation (b), Mini Batch K-Means demonstrates accelerated computation at the cost of edge artifacts (c), while MeanShift displays adaptive density recognition with boundary ambiguities (d). Visual comparison confirms DBSCAN (e) achieves optimal instance segmentation, characterized by well-defined cluster boundaries, natural preservation of insect-damaged cavities, and precise delineation of leaf vein branching patterns through spatially compact yet distinct clusters.

### 3.3 Investigation of neighborhood radius parameter ϵ on cotton leaf instance segmentation

The neighborhood radius parameter ϵ in the DBSCAN algorithm critically influences clustering efficacy. This experiment investigates the clustering performance of DBSCAN on cotton leaf point clouds under varying ϵ values, aiming to understand how parameter adjustments affect clustering precision and instance segmentation quality. The optimal value of ϵ=0.04 was determined through a systematic empirical process. We performed a grid search by incrementally increasing ϵ from 0.01 to 0.07 with a step size of 0.01. The evaluation metrics (ARI, NMI, FMI) showed a consistent trend: performance improved gradually as ϵ increased from 0.01, reached an optimal plateau around ϵ=0.04, and then began to decline as ϵ increased further beyond 0.05. This pattern confirms the existence of a well-defined optimum and underscores the sensitivity of the algorithm to this parameter. The value ϵ=0.04 was selected as it yielded the best overall performance across our dataset, effectively balancing the trade-off between over-segmentation in dense regions and under-segmentation in sparse regions.

However, it is important to note that this value is dataset-specific. Its efficacy relies on the consistent point cloud density and spatial scale of the plants in our current study. Variations in plant size, growth stage, or data acquisition protocols that alter the point cloud’s inherent density distribution would necessitate a re-calibration of this parameter for optimal results. Such analysis provides fundamental guidance for parameter selection in cotton leaf point cloud processing to achieve enhanced leaf recognition and feature extraction. The instance segmentation results of DBSCAN under different ϵ configurations are detailed in **Table 3**:

**Table 3:** Effectiveness comparison of DBSCAN instance segmentation with varying ϵ.

Neighborhood radius parameter ϵ	ARI	AMI	NMI	FMI
0.02	0.634	0.665	0.668	0.727
0.03	0.895	0.944	0.945	0.907
0.04	0.983	0.992	0.992	0.986
0.05	0.981	0.991	0.991	0.984
0.06	0.976	0.990	0.990	0.980
0.07	0.974	0.989	0.989	0.978

As evident from **Table 3**, the DBSCAN algorithm achieved optimal instance segmentation performance on cotton leaf point cloud data when the neighborhood radius parameter ϵ was set to 0.04, with all evaluation metrics approaching their peak values. As ϵ increased, the algorithm’s performance initially improved before marginally declining, demonstrating the significant impact and sensitivity of parameter selection on clustering outcomes.

**Figure 8:**
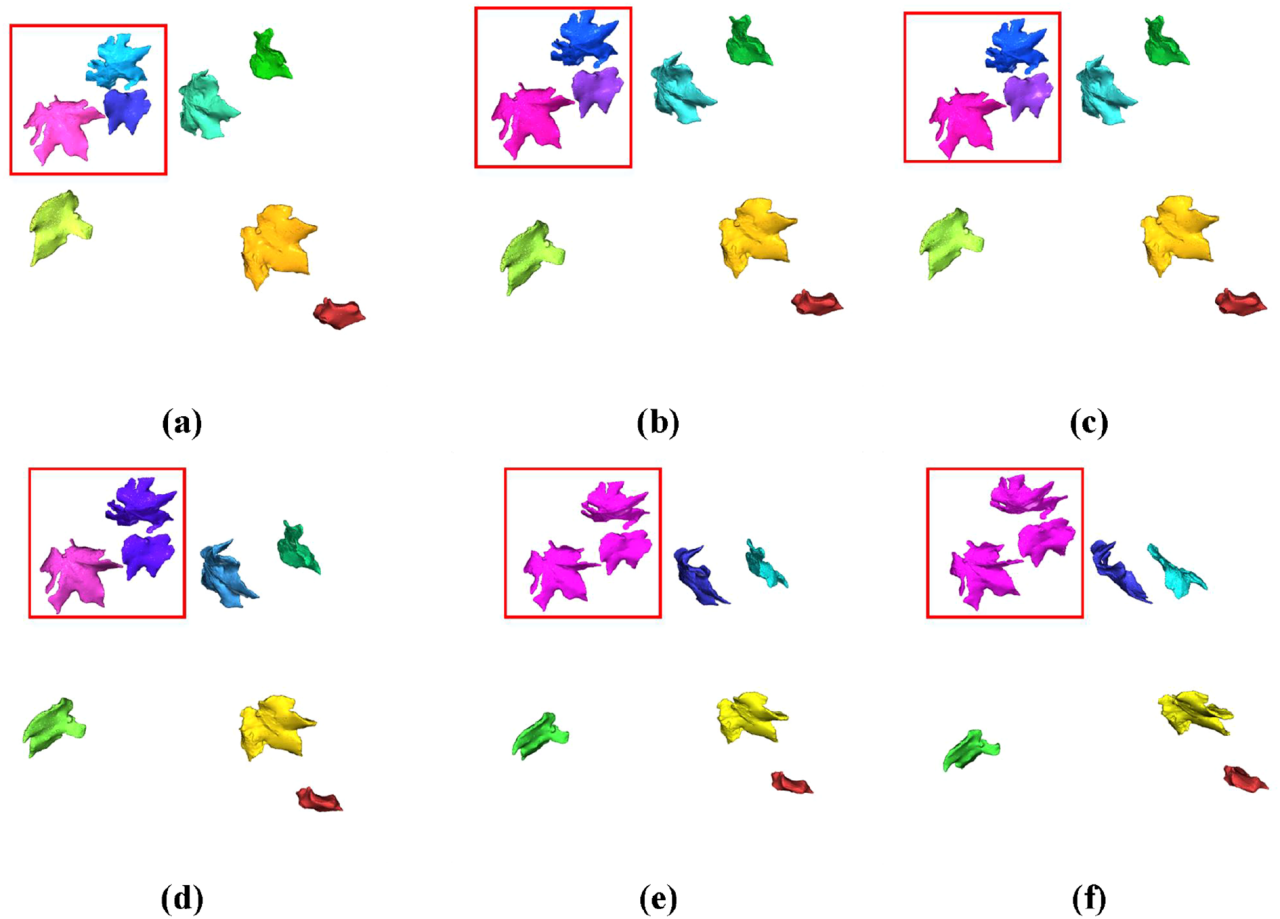
Parameter comparison for DBSCAN clustering (sparse leaf arrangement). **(a)** DBSCAN with the minimum neighborhood radius (ϵ=0.02); **(b)** DBSCAN with a reduced neighborhood radius (ϵ=0.03); **(c)** DBSCAN with the recommended neighborhood radius (ϵ=0.04); **(d)** DBSCAN with an increased neighborhood radius (ϵ=0.05); **(e)** DBSCAN with a high neighborhood radius (ϵ=0.06); **(f)** DBSCAN with the maximum neighborhood radius (ϵ=0.07).

**Figure 9:**
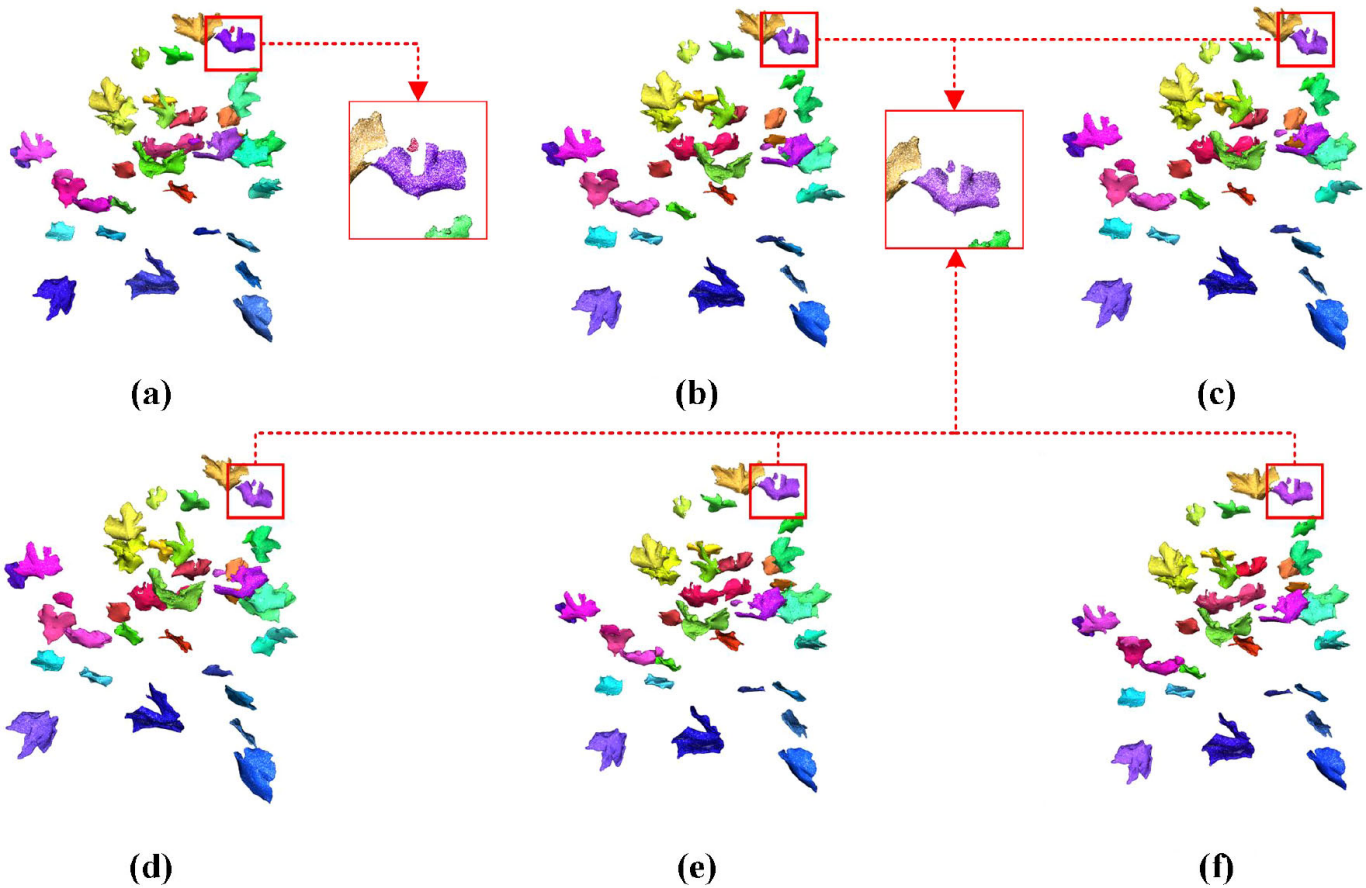
Parameter comparison for DBSCAN clustering (dense leaf arrangement). **(a)** DBSCAN with the smallest neighborhood radius (ϵ=0.02); **(b)** DBSCAN with a small neighborhood radius (ϵ=0.03); **(c)** DBSCAN with the optimal neighborhood radius (ϵ=0.04); **(d)** DBSCAN with a moderate neighborhood radius (ϵ=0.05); **(e)** DBSCAN with a large neighborhood radius (ϵ=0.06); **(f)** DBSCAN with the largest neighborhood radius (ϵ=0.07).

Two test samples were selected from the cotton point cloud dataset to visualize segmentation results, as shown in **Figures 8**, **9**.

In both figures, subfigures (a)-(f) correspond to neighborhood radius parameter settings ranging from 0.02 to 0.07. As shown in **Figures 8**, **9**, under dense leaf arrangements, lower ϵ values yield better instance segmentation efficacy for cotton leaves. Conversely, with sparser leaf distributions, higher ϵ values achieve optimal segmentation performance. When ϵ was set to 0.04, the algorithm demonstrated superior generalizability by maintaining relatively high segmentation accuracy across both dense and sparse leaf arrangements.

## 4 Discussion

### 4.1 Analysis of segmentation performance and research implications

This study successfully addresses the technical challenges of cotton plant point cloud leaf segmentation by introducing a two-stage segmentation method that integrates the PointNeXt deep learning framework with the DBSCAN density clustering algorithm. To clarify the contribution of this research, we note that similar deep learning approaches have been validated in phenotyping analyses of different crops. For instance, a recent study on apple trees also utilized PointNeXt to achieve high-precision semantic segmentation of branches and trunks (mIoU of 0.9481) (Jiang et al., 2025). That study demonstrated the effectiveness of the method in parsing the architecture of woody perennial fruit trees, while this research validates and extends this technical pathway to annual field crops, specifically targeting the more complex and variable organ structures of cotton leaves. This cross-crop comparison underscores the robustness and adaptability of point cloud deep learning in agricultural phenotyping.

When directly compared with contemporary research on cotton, our method demonstrates distinct advantages. For example, although Song et al (Jiazheng et al., 2024). employed Laplacian skeleton extraction combined with Quickshift++ for cotton plant organ segmentation, our approach achieved a higher mean intersection over union (mIoU) of 0.9846 and an accuracy of 0.9537 in the semantic segmentation task. This performance highlights the powerful capability of deep feature learning in parsing complex plant structures, particularly in distinguishing fine leaf veins and wrinkled edges—a challenge acknowledged in prior point cloud segmentation studies. For instance segmentation, the optimized DBSCAN algorithm (ϵ=0.04) exhibited excellent adaptability to non-uniform leaf distributions, achieving a high adjusted Rand index (ARI) of 0.983 and normalized mutual information (NMI) of 0.992. The end-to-end inference pipeline required only 0.88 seconds per sample, establishing a solid foundation for large-scale, high-throughput phenotyping applications and confirming its practical value for automated analysis.

### 4.2 Limitations and future perspectives

The primary application value of this research lies in its contribution to non-destructive, automated phenotypic analysis. Our framework enables the accurate extraction of key morphological parameters—such as leaf count, unfolding angle, and main stem height—replacing time-consuming and subjective manual measurements. This capability is crucial for precise growth monitoring and lays a foundation for future applications in stress resistance evaluation, for instance, by automating the calculation of metrics like wilted leaf area to support breeding decisions.

Despite these promising results, this study acknowledges certain limitations that present avenues for future work. First, the model was primarily trained and validated on data from controlled environments. Plant architecture in open fields is considerably more complex due to factors such as planting density, wind, pest/disease interactions, and varied lighting conditions. Therefore, the model’s performance may degrade under such unconstrained field conditions, representing a major constraint for large-scale practical deployment. Second, manual point cloud annotation remains a bottleneck for data production efficiency. Third, a critical technical limitation concerns the fixed neighborhood parameter in the DBSCAN algorithm, which caused approximately 7.1% over-segmentation in regions with extremely high point density (e.g., near the main stem). This issue arises because a static parameter cannot adapt to spatial density variations within the complex plant architecture.

To address these challenges and advance the methodology, future research will be guided by insights from several cutting-edge studies. To enhance field robustness, we will prioritize expanding the dataset to include point clouds of plants exposed to major agronomic stresses through controlled experiments and collaborative field collections. Such data would be instrumental in developing models capable of segmenting organs and quantifying stress-induced phenotypic changes. To overcome the limitation of fixed DBSCAN parameters, we will explore adaptive clustering approaches such as Hierarchical DBSCAN (HDBSCAN), which can dynamically adjust based on local density variations. Inspired by the homology constraint employed in HCMPE-Net for enforcing feature consistency in complex structures (Xiang et al., 2025), we plan to integrate constraints on leaf curvature and normal vector consistency to further mitigate over-segmentation. Furthermore, the hypergraph-based attention mechanism of the Hypergraph BiFormer (Jing et al., 2025) offers a promising path to better model the complex, non-pairwise spatial relationships between leaves in dense canopies. To specifically enhance the model’s robustness and adaptability in real-world field applications, the joint learning framework of SSC-Net (Sha et al., 2025a), which simultaneously handles segmentation and classification, provides a valuable blueprint for introducing auxiliary tasks such as stress state classification. Finally, to alleviate the burden of manual annotation and enhance data efficiency, techniques from small-sample learning models like ZHPO-LightXBoost (Sha et al., 2025c) will be explored to reduce reliance on massive labeled datasets, potentially combined with AI-assisted pre-labeling strategies.

## 5 Conclusions

This study developed and validated a novel two-stage segmentation method that effectively integrates the PointNeXt deep learning network and the DBSCAN clustering algorithm for cotton plant point cloud leaf segmentation. The framework demonstrated outstanding performance in both semantic and instance segmentation tasks, establishing a robust technical foundation for high-throughput phenotyping applications.

The main conclusions of this study are summarized as follows: First, the proposed method exhibits high accuracy and efficiency in segmenting cotton plant point clouds, with PointNeXt achieving an mIoU of 0.9846 in semantic segmentation and DBSCAN achieving an ARI of 0.983 in instance segmentation. Second, compared to existing methods for cotton organ segmentation, this study demonstrates superior performance in semantic segmentation, and its successful application to cotton extends the validation of point cloud deep learning from woody fruit trees to annual field crops. Third, this research provides a reliable technical basis for automating the extraction of key phenotypic traits, promoting the development of non-destructive and efficient crop phenotyping. Finally, the study also acknowledges limitations including reliance on manual annotation, over-segmentation issues, and primary validation in controlled environments.

Future research will focus on three key directions: First, Field Validation and Adaptation: Priority will be given to testing and optimizing the model on cotton plants grown in open fields to assess its robustness under real and complex agricultural conditions. Second, Advanced Synthetic Data Generation: Plans include developing a physics engine-based virtual point cloud generation system to create large-scale, accurately annotated training data that simulates field variability and various stress conditions. Third, Enhanced Segmentation Framework: Integrating geometric constraints with the deep learning framework to establish a semantics-geometry dual-driven adaptive segmentation framework, improving parsing accuracy in overlapping regions and under challenging field conditions.

This work pioneers a valuable technical pathway for 3D phenotypic analysis of cotton. The tools and insights provided can advance breeding programs and precision agriculture practices. By addressing the future directions outlined above, the potential of this method to create a tangible impact in real-world agricultural scenarios can be fully realized.

## Data Availability

The raw data supporting the conclusions of this article will be made available by the authors, without undue reservation.
